# Recent Advances in Intranasal Liposomes for Drug, Gene, and Vaccine Delivery

**DOI:** 10.3390/pharmaceutics15010207

**Published:** 2023-01-06

**Authors:** Van-An Duong, Thi-Thao-Linh Nguyen, Han-Joo Maeng

**Affiliations:** College of Pharmacy, Gachon University, 191 Hambakmoe-ro, Yeonsu-gu, Incheon 21936, Republic of Korea

**Keywords:** liposome, intranasal, systemic, brain, phospholipid, bioavailability, nose-to-brain, vaccine, immune, antibody

## Abstract

Liposomes are safe, biocompatible, and biodegradable spherical nanosized vesicles produced from cholesterol and phospholipids. Recently, liposomes have been widely administered intranasally for systemic and brain delivery. From the nasal cavity, liposome-encapsulated drugs and genes enter the systemic circulation primarily via absorption in the respiratory region, whereas they can be directly transported to the brain via the olfactory pathway. Liposomes can protect drugs and genes from enzymatic degradation, increase drug absorption across the nasal epithelium, and prolong the residence time in the nasal cavity. Intranasal liposomes are also a potential approach for vaccine delivery. Liposomes can be used as a platform to load antigens and as vaccine adjuvants to induce a robust immune response. With the recent interest in intranasal liposome formulations, this review discusses various aspects of liposomes that make them suitable for intranasal administration. We have summarized the latest advancements and applications of liposomes and evaluated their performance in the systemic and brain delivery of drugs and genes administered intranasally. We have also reviewed recent advances in intranasal liposome vaccine development and proposed perspectives on the future of intranasal liposomes.

## 1. Introduction

Nanotechnology has had a profound impact on pharmaceutical sciences, primarily due to the development of nanosized drug delivery systems in the last few decades [1,2,3]. Nanoparticles (typically <1000 nm) loaded with drug or gene molecules have many advantages, such as their high surface area and improved solubility, absorption, and stability [4,5]. Many studies have focused on liposomes, which are spherical nanosized vesicles similar to biological membranes that are produced from cholesterol and phospholipids [6,7]. Liposomes are highly safe, biocompatible, and biodegradable; thus, they are ideal carriers for various drug delivery routes [8]. At least 16 liposome-based products are presently being used, primarily for intravenous (IV) and intramuscular (IM) injections [9,10]. For example, Doxil (doxorubicin) was the first liposome-based product (and also the first nano-drug) approved by the United States Food and Drug Administration (US FDA) in 1995 for the treatment of certain cancers, including ovarian cancer and AIDS-related Kaposi’s sarcoma [11]. In addition, other liposome-based products, such as DaunoXome (daunorubicin), Myocet^®^ (doxorubicin), AmbiSome (amphotericin B), and Onivyde™ (irinotecan), are available for clinical use [10]. In 2018, Arikayce (amikacin liposome inhalation suspension) was approved to treat *Mycobacterium avium*-mediated lung disease.

**Figure 1:**
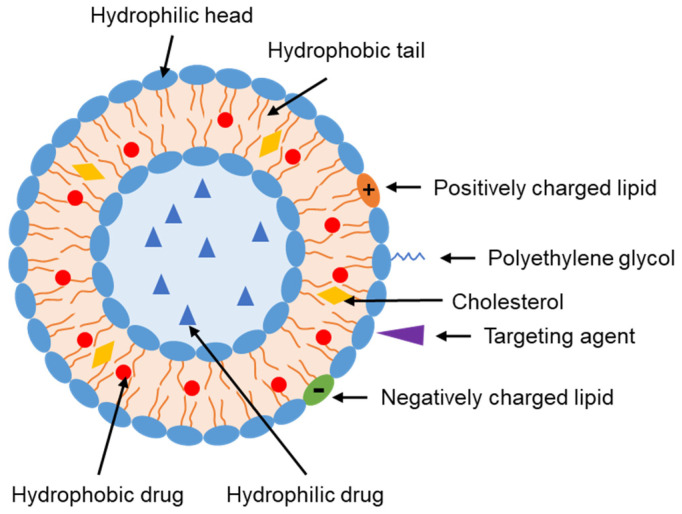
Liposome structure and some modifications. Targeting agents include peptides, proteins, carbohydrates, small molecules, ligands, aptamers, and antibodies.

Liposomes were first developed as artificial lipid vesicles by Bangham et al. in 1965 [12] and since then they have been extensively employed in drug and nutrition delivery [13]. They comprise lipid bilayers and can load both hydrophilic molecules (in the water-soluble core) and hydrophobic molecules (in bilayer membranes) (Figure 1) [14]. Liposomes are produced from phospholipids, which are amphiphilic molecules comprising a hydrophilic head and hydrophobic tails. Liposomes may be neutral, cationic, or anionic, depending on the constituting phospholipids [15,16]. The neutral phospholipids include phosphatidylcholine, phosphatidic acid, and phosphatidylethanolamine. The typical negatively charged phospholipids include phosphatidylglycerol and phosphatidylserine (PS). Incorporating positively charged lipids such as N-[1-(2,3-dioleyloxy)propyl]-N,N,N-triethylammonium (DOTMA) and 1,2-dioleoyl-3-trimethylammoniopropane (DOTAP) can form positively charged liposomes, which are widely used in gene delivery, owing to the interaction between these lipids and the negatively charged nucleic acids [17]. Cationic liposomes are also used for protein delivery because of their higher protein encapsulation efficiency and better cellular uptake than anionic liposomes [18]. Sterols (such as cholesterol) are also incorporated into liposomes. Cholesterol molecules are distributed in phospholipid bilayers with their hydroxy groups close to the hydrophilic heads and their aromatic rings are aligned with the hydrophobic tails. This cholesterol configuration can modulate liposome rigidity [19], stability [20], fluidity, and permeability [21]. Various molecules can be used to functionalize the surface of liposomes to obtain specific properties. For example, polyethylene glycol (PEG) has been used to produce long-circulating liposomes. Peptides, proteins, carbohydrates, small molecules, ligands, aptamers, and antibodies can also be incorporated to target solid tumors. Stimuli-responsive liposomes (such as pH-, temperature-, and hypoxia-responsive liposomes) have been developed to enhance the delivery of anticancer agents at the tumor site [22].

In recent years, intranasal (IN) administration of nanosized drug delivery systems has been intensively investigated [23,24]. IN administration is a conventional noninvasive drug delivery approach for treating local nasal diseases such as infectious rhinitis, nasal polyposis, and sinusitis [25]. However, drugs and genes administered intranasally can also enter the systemic circulation and brain, making IN administration a potential approach for systemic and brain delivery of various drugs and genes [26]. Drugs and genes can be absorbed from the nasal cavity into the bloodstream or transported to the brain via nose-to-brain pathways that bypass the blood–brain barrier (BBB). IN administration is a practical strategy for vaccine delivery [27]. IN vaccination, which can elicit both systemic and mucosal immunities, is a noninvasive approach for mass vaccination in large populations, including elderly patients and children [28]. It is particularly appropriate to halt the progression of respiratory diseases (such as the coronavirus disease, 2019 (COVID-19), caused by severe acute respiratory syndrome coronavirus 2 (SARS-CoV-2)), since the nasal compartment is the first point of virus entry before they spread to the lungs [29].

Many nanosized drug delivery systems developed recently, such as micelles [30], nanoemulsions [31,32], solid lipid nanoparticles [33], nanostructured lipid carriers [34,35], polymeric nanoparticles [36], and liposomes [37], have been administered intranasally due to the long residence times in the nasal cavity, improved stability, and increased uptake. Liposomes are a promising platform for drug, gene, and antigen delivery. IN liposomes can improve the accumulation in the brain of small molecules [38,39], peptides [40], proteins [18], and nucleic acids [41], as well as the systemic exposure to different molecules [40,42,43,44]. IN liposomes are also a potential approach for vaccine delivery, with the advantages of high patient compliance and low infection risk [45,46,47]. Cationic liposomes can be used to deliver vaccine adjuvants to enhance the immune response. Considering the recent interest in IN liposomes, this review aims to summarize the latest progress in liposome development for the IN delivery of drugs, genes, and vaccines. First, we discuss the properties of IN administration and liposomes that are beneficial for IN administration for systemic and brain delivery. We discuss not only recent formulation advancements and applications of liposomes but also evaluate their performance in drug, gene, and vaccine delivery.

## 2. Liposomes: Classification, Preparation, Characterization, and Properties

### 2.1. Liposome Classification

Liposomes can be classified according to their structure, physicochemical properties, or in vivo fate. Depending on the phospholipid used, liposomes can be neutral, cationic, or anionic [15,16]. Based on the lamellae, they can be unilamellar, oligolamellar, or multilamellar liposomes. Unilamellar liposomes can be further classified into small unilamellar liposomes (SUV, <200 nm) and large unilamellar liposomes (>200 nm) [48]. The SUVs exhibit the enhanced permeation and retention (EPR) effect (the ability to progressively accumulate in the tumor vasculature) and thus, are of interest for anticancer drug delivery [49,50,51]. Multi-lamellar liposomes (MLVs) generally show high integrity and prolonged drug circulation; however, they cannot circulate in the blood, cross biological barriers, or improve cellular uptake of drugs [48].

The in vivo biological fates of both conventional and long-circulating liposomes have been studied. After entering the body, conventional liposomes rapidly accumulate in the liver and spleen because they are recognized by the reticuloendothelial system (RES) [52]. On the other hand, the surface of long-circulating liposomes can be modified with a polymer (such as a PEG), which protects the liposomes from RES, reduces their renal clearance, prolongs circulatory time, and increases bioavailability. Consequently, they can reduce dose frequency, toxicity, and side effects [51]. They are also called stealth liposomes and have been used in Doxil and Lipidox (doxorubicin). However, PEGylation may generate anti-PEG antibodies, which increases liposome elimination [53]. Thus, other polymers, such as polysialic acid, have been used to prepare long-circulating liposomes [54].

### 2.2. Preparation Methods

Various methods have been used to produce liposomes. In this section, the most common and widely used methods are discussed. Thin-film hydration is the oldest and most straightforward method for preparing liposomes. This method comprises three main steps: (i) dissolution of lipids and drugs in an organic solvent, (ii) solvent evaporation (generally by rotary evaporation) to form a dried thin lipid film, and (iii) film hydration with an aqueous solution to form liposomes [55]. In this method, lipophilic drugs can be dissolved in an organic solvent together with phospholipids, whereas hydrophilic molecules can be dissolved in an aqueous solution and then incorporated into liposomes during the hydration process. The disadvantages of this method are its low entrapment efficiency, difficult removal of organic solvents, and small-scale production [56].

In the ethanol injection method, an ethanol solution containing lipids and drugs is injected into an aqueous phase using a syringe. It is then evaporated by mechanical stirring [57,58]. The rapid dilution of ethanol upon injection in the aqueous phase results in the formation of bilayer planar fragments due to lipid precipitation. Subsequently, the lipid fragments fuse to form closed unilamellar vesicles after removing the ethanol. This method has also been used to prepare solid lipid nanoparticles and nanostructured lipid carriers [59]. The ethanol injection method is simple, reproducible, easy to scale up, and less toxic. However, this method involves a large amount of the aqueous phase (leading to strongly diluted liposomes), and solvent removal is difficult. Some factors, such as ethanol volume, injection speed, and temperature, must be carefully controlled during the production process [58].

Reverse-phase evaporation has been used in several studies. Lipids are first dissolved in an organic solvent mixture (such as diethyl ether/chloroform, diethyl ether/isopropyl ether, and chloroform/methanol) to form inverted micelles [60]. An aqueous phase is then added to the organic phase under mechanical stirring or sonication to facilitate water-in-oil microemulsion formation. The organic solvent is subsequently removed by rotary evaporation. During this process, a viscous gel temporarily forms before some droplets are disrupted. The phospholipids are then distributed around the water-in-oil microemulsion to form liposomes [61]. This method enables the encapsulation of large amounts of macromolecules [62]. However, its limitations include the presence of residual solvents, difficulties in scaling up, and drug exposure to organic solvents. This method is unsuitable for biomolecules such as enzymes, proteins, and oligonucleotides [63].

After liposomes are formed, some techniques, such as sonication [64], extrusion [65], and high-pressure homogenization [66], can be employed to reduce their size. Sonication includes bath and probe sonication. This is one of the most common approaches for producing SUVs [67]. However, it can also cause lipid degradation and metal contamination. The extrusion method is also widely used. In this method, liposomes are passed through a membrane (25 nm to 1 mm) several times. This downsizing method is highly reproducible. However, its limitations include product loss and small-scale production [68,69]. The high-pressure homogenization method reduces liposome size due to cavitation, shear phenomena, and turbulence. This method may lead to broad liposome size distribution and metal contamination [66].

During liposome preparation, drugs can be dissolved in an organic or aqueous phase depending on their hydrophilicity [55]. For example, water-soluble donepezil is dissolved in a phosphate buffer for film hydration [39], whereas water-insoluble risperidone is dissolved in an organic phase [70]. In gene delivery, to improve the encapsulation of negatively charged nucleic acids in the liposome core, positively charged lipids, such as DOTMA and DOTAP, are generally incorporated [17]. In addition, co-encapsulation with a cationic peptide is also applied to compact nucleic acids by electrostatic interaction in the liposome core [41,71]. In vaccine delivery, blank liposomes can be prepared beforehand, and antigens are then adsorbed onto the liposome surface [72,73,74]. Alternatively, antigens can be encapsulated during liposome preparation by dissolving them in an organic [75,76] or aqueous phase [77,78,79].

### 2.3. Liposome Characterization

#### 2.3.1. Physicochemical Characterization

The size distribution of liposomes can be measured using dynamic light scattering, where fluctuations in the scattered light intensity are affected by the particle hydrodynamic radius. Both particle size and polydispersity index are used to evaluate the liposomes. Liposomes with diameter <150 nm are favorable to target tumors because they can escape from the blood vessel capillaries and enter the tumor environment. Liposomes with diameter <100 nm can avoid immune system phagocytosis clearance and circulate longer in the blood stream [80]. Some studies have found that liposome size does not affect immunization when administered intranasally [72,81]. The polydispersity index indicates the size distribution of liposomes. The preferable polydispersity index for most liposome dispersions is <0.3, which indicates a monodispersed system [82].

The zeta potential of liposomes is their electric potential, which can also be measured using dynamic light scattering [82]. This value reflects ionization or ion adsorption of the lipid heads of the liposomes. Zeta potential affects the colloidal stability, absorption, biodistribution, and pharmacokinetics (PK) of liposomes. Liposomes with a high zeta potential exhibit strong repulsion between particles, which reduces particle aggregation. On the other hand, a low zeta potential indicates weak repulsion, which can be exceeded by attraction and consequently cause the flocculation or coagulation of liposomes. Entrapment efficiency is the ratio of the drug or gene entrapped in the liposomes to the total amount used in the preparation. Entrapment efficiency is usually reported for small-molecule drugs [67,83], peptides [40], and proteins [84] encapsulated in liposomes.

Other techniques have been used to characterize liposomes as well as other colloidal systems. The structural properties of liposomes are characterized using small-angle X-ray scattering, wide-angle X-ray scattering, small-angle neutron scattering, and X-ray diffraction. Other methods, such as infrared spectroscopy, differential scanning calorimetry, and nuclear magnetic resonance, are helpful in studying drug–lipid interactions. High-resolution visualization of liposomes can be achieved using scanning electron microscopy, transmission electron microscopy, and atomic force microscopy [63,85].

#### 2.3.2. Pharmacokinetic Evaluation

In addition to the above-mentioned physicochemical analyses, the pharmacokinetic characteristics of liposomes, such as in vitro BBB permeation [67], ex vivo nasal tissue permeation [38,77], cellular uptake [75], cytotoxicity [78], and histopathological examinations [40,43], have been characterized. The immune responses of IN-liposome vaccines in animal models or humans are reflected by the immunoglobulin G and A (IgG and IgA) levels. IgG is the most common antibody released by plasma B cells in the blood. In some cases, the levels of IgG1 and IgG2a (IgG subclasses) are also measured [86]. IgA levels are usually determined in nasal, lung, and vaginal tissues [86]. PK and biodistribution studies are generally performed to evaluate IN liposomes for drug and gene delivery. IN liposomes can be compared with IV liposomes, IN or IV-free drugs, or IN, IV, or oral commercial dosage forms. In PK studies, the maximum drug concentration (C_max_) and area under the concentration–time curve (AUC_plasma_ and AUC_brain_) were calculated and compared among formulations. Different parameters to evaluate the brain-targeting efficacy of the liposomes, such as drug targeting efficiency (DTE), drug transport percentage (DTP), B_IN/IV_, and RB, are calculated as follows [87,88].

(1) $$\mathrm{DTE}=\frac{\left({\mathrm{AUC}}_{\mathrm{brain},\mathrm{IN}}\right)/\left({\mathrm{AUC}}_{\mathrm{plasma},\mathrm{IN}}\right)}{\left({\mathrm{AUC}}_{\mathrm{brain},\mathrm{IV}}\right)/\left({\mathrm{AUC}}_{\mathrm{plasma},\mathrm{IV}}\right)}$$

(2) $$\mathrm{DTP}=\frac{{\mathrm{AUC}}_{\mathrm{brain},\mathrm{IN}}-\frac{{\mathrm{AUC}}_{\mathrm{brain},\mathrm{IV}}}{{\mathrm{AUC}}_{\mathrm{plasma},\mathrm{IV}}}\times {\mathrm{AUC}}_{\mathrm{plasma},\mathrm{IN}}}{{\mathrm{AUC}}_{\mathrm{brain},\mathrm{IN}}}=1-\frac{1}{\mathrm{DTE}}=1-\frac{100}{\mathrm{DTE}\%}$$

(3) $$B_{\mathrm{IN}/\mathrm{IV}}=\frac{{\mathrm{AUC}}_{\mathrm{brain},\mathrm{IN}}}{{\mathrm{AUC}}_{\mathrm{brain},\mathrm{IV}}}$$

(4) $$\mathrm{RB}=\frac{{\mathrm{AUC}}_{\mathrm{brain},\;\mathrm{liposomes}\;\left(\mathrm{IN}\right)}}{{\mathrm{AUC}}_{\mathrm{brain},\mathrm{free}\;\mathrm{drug}\;\left(\mathrm{IN}\right)}}$$

These values are typically expressed as percentages. DTE is used to assess whether IN liposomes target the brain better than free drug or IV liposomes. DTE% > 100 indicates efficient brain targeting. DTP values indicate the proportion (or percentage) of drugs entering the brain via the olfactory and trigeminal pathways (direct routes). The subtraction in Equation (2) indicates the amount of drug entering the brain from the systemic circulation (across the BBB). DTP% ranges from 0 to 100. DTP% = 0 suggests no nose-to-brain delivery via the direct route, whereas DTP% = 100 indicates no drug crossing the BBB to enter the brain. Higher DTE and DTP for IN liposomes than those for IN-free drugs can be used to demonstrate the critical role of liposomes in nose-to-brain drug delivery [88]. B_IN/IV_ indicates the ratio of IN-liposome drug accumulation to IV-free drugs or liposome accumulation in the brain. RB is the ratio of IN-liposome drug accumulation to IN-free drug accumulation in the brain. B_IN/IV_ and RB reflect the absolute increase in drug accumulation in the brain via IN administration of liposomes [89]. Many studies have reported the enhanced AUC_plasma_ or AUC_brain_ of IN liposomes. For example, AUC_plasma_ and AUC_brain_ of IN liposomes were higher than those of IN-free donepezil [39] and rivastigmine [43]. In some studies, IN liposomes had high DTE and DTP values, indicating nose-to-brain transport, such as DTE = 2903–3870% and DTP = 95.6–97.4% for baicalin-loaded liposomes [90] and DTE = 302.22% and DTP = 63.66% for cyclovirobuxine D-loaded liposomes [67].

In biodistribution studies, drug accumulation in different organs (such as the brain, liver, spleen, kidneys, and lungs) was determined. The accumulation of brain-targeted liposomes should increase in the brain and decrease in other untargeted organs. Fluorescence imaging [37], isotope labeling [91,92], and green fluorescent protein (GFP) [68] have been used to visualize and confirm liposome delivery to the olfactory bulb and different parts of the brain (such as the striatum, cortex, and cerebellum) after IN administration. For example, successful brain delivery of cationic liposomes containing GFP mRNA and luciferase mRNA was confirmed by GFP fluorescence [68]. Alternatively, the brain or its regions can be collected to determine the drug levels and calculate the AUC in the brain. For example, IN liposomes showed better rivastigmine distribution in the hippocampus and cortex than IN and IV-free drugs [93]. Pharmacodynamic studies have been performed to evaluate the efficacy of IN liposomes using different animal models depending on the disease [56,90,94].

### 2.4. Liposome Stability

One of the major limitations of liposomes is their poor physical and chemical stability. Liposome stability is affected by several factors, such as composition, phospholipid:cholesterol ratio, fatty acid side chains, unsaturation degree, polar head chemistry, and chain length [95]. Because of the electrostatic effect among liposome particles, liposomes may aggregate during storage, leading to increases in vesicle size and drug loss. The physical stability of liposomes can be improved by increasing electrostatic repulsion by increasing their surface charge. Liposomes can be chemically degraded through ester bond (between glycerol and fatty acids) hydrolysis and unsaturated acyl chain peroxidation, which remarkably reduces their quality and stability [95]. Therefore, it is preferable to transform them into solid form by lyophilization [96] or spray drying [97].

In a previous study, liposomes loaded with ghrelin, a cationic peptide hormone, were transformed into dried powder by spray drying, which substantially increased their size, PDI, and zeta potential (195 nm to 263 nm, 0.082 to 0.203, and +5 mV to +9 mV, respectively) [98]. Despite that, compared to the liposome suspensions, the powder exhibited desirable properties, including stronger adhesion to mucins (89% vs. 61%), higher protection against trypsin (26% vs. 20%), and lower peptide storage degradation at 25 °C after 4 weeks (2.67% vs. 95.64%).

## 3. Intranasal Liposomes for Systemic and Brain Delivery of Drugs and Genes

### 3.1. Drug and Gene Transport following Intranasal Administration

The human nasal cavity is divided into vestibule, olfactory, and respiratory regions [99]. The vestibule region is the frontal part of the nasal cavity, which has nasal hairs to filter air and prevent the entrance of large particles. This region is less vascularized; thus, it is not considerably involved in drug absorption in IN delivery [100]. The olfactory region is the upper part of the nasal cavity, with a surface area of ~10 cm^2^ [99]. This region has olfactory nerves connecting olfactory epithelia and olfactory bulb, which play a critical role in direct nose-to-brain drug and gene transport [101]. The respiratory region is the largest area of the nasal cavity (~160 cm^2^) and is highly vascularized [99]. Therefore, drug and gene absorption into the blood from the nasal cavity primarily occurs in this respiratory process [102]. The respiratory epithelium in this region comprises basal cells, mucus-secreting goblet cells, and ciliated and non-ciliated columnar cells [99]. Some respiratory cells are covered with cilia, which play a primary role in mucociliary clearance that protects the respiratory tract from pathogens or particles [100]. In this region, the ophthalmic and maxillary branches of the trigeminal nerves are responsible for the transport of drugs and genes directly to the brain [87].

**Figure 2:**
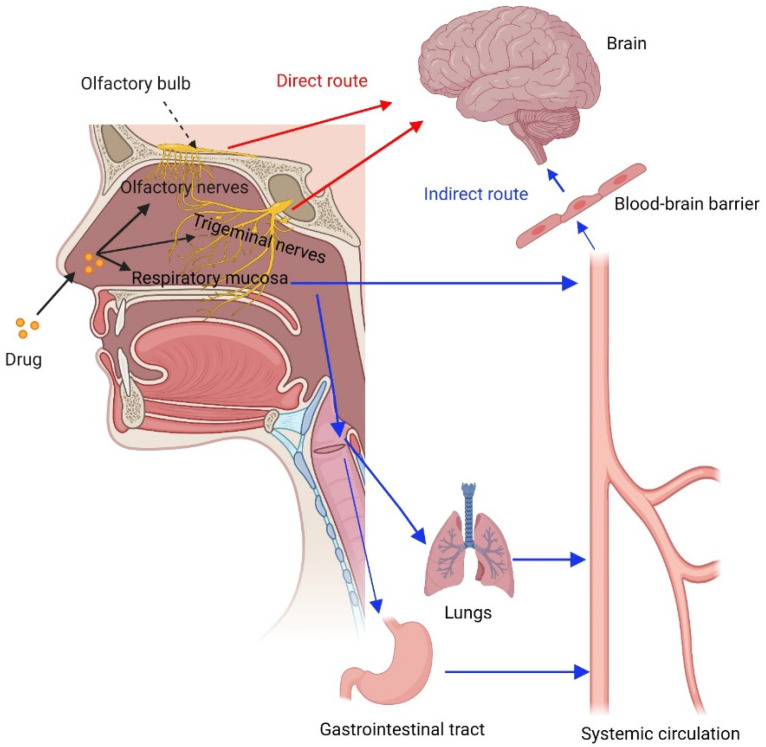
Transport routes of drugs from the nose to the systemic circulation and the brain.

Following IN administration, drugs and genes can be absorbed into the systemic circulation in the respiratory region of the nasal cavity [103]. Systemic absorption can also occur via other routes, such as the lymphatic system, oral cavity, gastrointestinal tract, and lungs [47]. Simultaneously, there is direct nose-to-brain transport to deliver drugs and genes into the brain via IN administration [104]. Drugs and genes in the nasal cavity are distributed to the olfactory region, from where they can be transported to the brain mainly via an extraneuronal route along olfactory neurons in only a few minutes. This is the primary mechanism of the nose-to-brain transport of IN drugs and genes [87,101]. Other mechanisms occurring in the olfactory region include intraneuronal olfactory neuron endocytosis, supporting cell endocytosis, and intercellular transport across tight junctions, which may be time-consuming [105]. Drugs and genes can also be transported to the brain via the trigeminal nerve pathway by the extraneuronal and intraneuronal routes [87]. There is also an indirect pathway to deliver drugs and genes to the brain, through which the drugs and genes first enter systemic circulation after IN administration, cross the BBB, and reach the brain. This mechanism is less critical because the BBB contains efflux pumps, such as P-glycoprotein, that prevent most molecules from entering the brain [26,106,107]. The drug and gene transport routes from the nasal cavity to the systemic circulation and the brain are illustrated in Figure 2.

### 3.2. Considerations for Intranasal Liposomes for Systemic and Brain Delivery

Drugs and genes entering the systemic circulation and brain via IN administration can bypass extensive first-pass metabolism (both intestinal and hepatic) and degradation by gastrointestinal fluids [108]. This route provides a quicker onset of action and fewer side effects than oral administration. This route is also feasible for some large molecules (such as peptides and proteins) that are not easily absorbed via the oral route. IN administration enhances patient compliance and allows self-administration [89]. However, IN formulations have short residence times in the nasal cavity since they are quickly cleared from the absorption site via mucociliary clearance. This limits the absorption time of drugs and genes into the systemic bloodstream and brain, resulting in low bioavailability in the nasal cavity [109].

These limitations can be overcome when drugs and genes are encapsulated in liposomes. Liposomes increase drug absorption across the nasal epithelium owing to the presence of permeation enhancers. Nanosized particles can enhance absorption via endocytosis or paracellular transport [109]. Liposomes also protect drugs and genes from enzymatic degradation. Liposomes can be decorated with mucoadhesive polymers (such as chitosan) or loaded into gels. Mucoadhesive polymers may interact with mucus via hydrogen and van der Waals bonds and electrostatic attraction, which reduce mucociliary clearance and prolong the residence time of IN liposomes [110,111]. Some typical mucoadhesive polymers are chitosan, chitosan derivatives, and conjugates, alginates, starch, gelatin, gums, methylcellulose, HPMC, carboxymethylcellulose, sodium hyaluronate, polyacrylates, polymethacrylates, and crospovidone [112,113,114]. For example, coating liposomes with chitosan increased the mucoadhesion of the liposomes and resulted in an increased AUC_plasma_ of sumatriptan [115]. Maleimide has been used in many studies to develop mucoadhesive liposomes due to its interactions with the sulfhydryl groups in mucins [116,117]. This polymer can be used for functionalizing IN liposomes with mucoadhesive characteristics in future studies. Liposomes can be functionalized with cell-penetrating peptides (CPPs) to increase their transport across absorption barriers [41,93]. Some gels have been used to minimize enzymatic degradation and mucociliary clearance and enhance nasal absorption of IN liposomes, such as thiolated chitosan [42] and gellan gum–xanthan gum gels [38]. In addition, other factors must be considered when developing IN liposomes. IN administration can only deliver a limited volume of formulations (<200 µL in humans); thus, this route is only suitable for low-dose drugs. In addition, the components, pH values, and viscosities of IN formulations must be compatible with the nasal mucosa to prevent irritation, destruction, and inflammation of the nasal epithelium. Nasal cavity conditions may affect drug and gene absorption, and alter therapeutic effects [47].

In the following sections, we discuss recent advances in the development of IN liposomes for the delivery of small-molecule drugs, therapeutic peptides, proteins, and nucleic acids.

### 3.3. Intranasal Liposomes Containing Small-Molecule Drugs

Donepezil is a cholinesterase inhibitor that increases acetylcholine levels in the brain to treat Alzheimer’s disease (AD). Donepezil was loaded into liposomes for IN administration [39]. In rats, the IN liposomes showed higher AUC_plasma_ (2.8- and 1.9-fold) and AUC_brain_ (1.7- and 1.4-fold) than the oral and IN-free drug, respectively. In another study, donepezil liposomes were developed and incorporated into a thiolated chitosan hydrogel, which increased the drug accumulation in the brain of rabbits (2.1-fold versus an oral tablet) [42]. The AUC_plasma_ also increased by 1.5-fold. Recently, Rajput et al. prepared donepezil liposomes and loaded them into an in situ gel for IN administration [38]. The IN liposome-based gel exhibited a 6.5-fold higher drug permeability than the IN donepezil HCl solution-based gel. In rats, the IN liposome-based gel showed a 2.2-fold higher AUC_brain_, 1.4-fold lower AUC_plasma_, and 12-fold lower liver accumulation of donepezil compared with an oral marketed gel. Furthermore, the IN liposome-based gel reversed the scopolamine-mediated alteration in acetylcholinesterase, sodium dismutase, catalase, glutathione, and malondialdehyde levels in amnesia rats (induced by scopolamine). Thus, the IN-donepezil liposome-based gel could be effectively transported into the brain while reducing systemic and liver accumulation. These characteristics are favorable for brain-targeted formulations.

Rivastigmine is another FDA-approved cholinesterase inhibitor recommended for AD treatment. It has low oral bioavailability due to its extensive first-pass metabolism and poor BBB penetration. Oral therapy also causes a high incidence of systemic adverse effects and unpredictable systemic exposure [118]. Therefore, IN liposomes have been developed to improve drug delivery to the brain. Yang et al. prepared rivastigmine-loaded liposomes modified with a CPP [93]. The IN liposomes showed better rivastigmine distribution in the hippocampus and cortex, as well as intense inhibition of acetylcholinesterase and butyrylcholinesterase activities in rats. El-Helaly et al. developed electrostatic stealth rivastigmine-loaded liposomes using didecyldimethylammonium bromide, a positive charge inducer, to increase their stability [43]. The AUC_plasma_ and AUC_brain_ of the IN liposomes in rabbits were 4.2- and 4.9-fold higher than those of the IN-free drug solution, respectively. In a recent study, the systemic bioavailability and clearance rate of rivastigmine-loaded liposomes increased (27-fold) and decreased (2-fold), respectively, compared with those of the oral free drug [56]. In rats with scopolamine-induced acute dementia or colchicine-induced chronic dementia, the drug-loaded liposomes significantly rescued memory deficits compared to the oral and IN-free drug solutions assessed in the Morris water maze and passive avoidance tasks. The limitation of this study is that the improved drug accumulation in the brain following IN administration of rivastigmine-loaded liposomes was not confirmed.

Galantamine, a phenanthridine alkaloid, is also an FDA-approved cholinesterase inhibitor used to treat AD. Since oral administration and injection of galantamine can cause side effects in the gastrointestinal tract and affect other tissues, the IN-administration route has been investigated to enhance brain delivery of this drug. Li et al. developed liposomes loaded with galantamine hydrobromide for nose-to-brain delivery [119]. In rats, IN liposomes exhibited higher acetylcholinesterase inhibition, C_max,brain_ (3.5-fold), and AUC_brain_ (3.4-fold) than the oral free drug. The results suggest some promise for IN galantamine liposomes in targeting the brain for AD treatment.

Other AD drugs have also been encapsulated in IN liposomes for delivery to the brain. Celecoxib, a cyclooxygenase-2 specific inhibitor, can exert neuroprotective effects in AD treatment. In a previous study, celecoxib was loaded into liposomes or erythrocyte membranes [94]. Following IN administration, both systems showed a higher brain distribution in mice than the IN-free drug suspension, inhibited β-amyloid deposition, and improved cognitive decline in APP/PS1 transgenic mice (mutations associated with early onset AD). The effects of concurrently increasing neurogenesis and decreasing apoptosis in erythrocyte membrane-based systems were better than those of liposomes. Imatinib is a tyrosine kinase inhibitor with anti-AD activity. Saka et al. previously developed IN liposomes loaded with imatinib mesylate, which showed approximately 7-fold higher AUC_brain_ than oral and IN-free drug solutions in rats [57]. Hydroxy-α-sanshool, an unsaturated fatty acid amide found in *Zanthoxylum bungeanum*, possesses anti-AD activity. In a recent study, Li et al. loaded this compound into liposomes to improve its stability and deliver it to the brain via IN administration [83]. The drug-loaded liposomes caused almost no damage to the nasal mucosa, indicating the safety of the developed liposomes. In mice with D-galactose-induced learning memory deficits, liposomes showed improved learning and memory abilities when tested with passive avoidance and Morris water maze tests. In the pathological assay, liposomes protected against neuronal cell damage. Furthermore, following IN administration, the AUC_plasma_ and AUC_brain_ of the liposomes were 1.7- and 2.1-fold higher than those of the free drug, respectively. Therefore, IN liposomes may be promising carriers for hydroxy-α-sanshool delivery to the brain to ameliorate learning and memory disorders in AD.

Rizatriptan is used for migraine management. Padalkar et al. developed rizatriptan-loaded liposomes with cholesterol glutathione conjugates because glutathione could function as a ligand to target drug delivery to the brain via the IN route [120]. They found that the IN-conjugated liposomes exhibited higher AUC_plasma_ (1.3- and 2.4-fold) and AUC_brain_ (3.4- and 6.7-fold) in rats compared with the IN-non-conjugated liposomes and oral tablet, respectively. In another study, rizatriptan was loaded into liposomes containing only lecithin [121]. In rats, IN liposomes exhibited increased AUC_plasma_ (4-fold) compared with the oral drug solution; however, the brain distribution was not investigated. Another drug used to treat migraine is sumatriptan. Sumatriptan-loaded liposomes were prepared and coated with chitosan to increase mucoadhesion for IN administration [115]. The IN chitosan-coated liposomes showed higher AUC_plasma_ than IN-uncoated liposomes and IN-free drug solution in rabbits.

IN liposomes have also been used to encapsulate other drugs used in different central nervous system disorders. Narayan et al. developed risperidone-loaded liposomes to deliver drugs to the brain via IN administration for the management of schizophrenia [70]. Three liposome formulations were evaluated: unmodified liposomes, cationic liposomes (with stearylamine), and PEGylated liposomes. All IN liposomes showed higher AUC_brain_ of risperidone in rats than the IV-free drug. The IN PEGylated liposomes exhibited the highest AUC_brain_ improvement (1.7-fold) and a DTE% of 120%. However, the DTP% for the PEGylated liposomes was 16.7%. Thus, most of the drug (83.3%) entered the brain by crossing the BBB after the liposomes were absorbed into the systemic circulation.

Wei et al. developed liposomes loaded with cyclovirobuxine D, a steroidal alkaloid used to treat cerebrovascular diseases and other cardiovascular disorders [67]. Angiopep-2 and Tween 80 were incorporated to increase brain delivery. The angiopep-2 Tween 80-coated liposomes (targeted liposomes) showed higher in vitro BBB transport of the drug than the nontargeted liposomes and free drug solution. In rats, the IN-targeted liposomes showed DTE% and DTP% of 302.22% and 63.66%, respectively, which were higher than those of the IN-free drug and IN-nontargeted liposomes. The AUC_brain_ for the targeted liposomes (IN) was 2.1-, 1.4-, and 1.9-fold higher than that of the IN-free drug, IN-nontargeted liposomes, and IV targeted liposomes, respectively. Thus, targeted liposomes (with angiopep-2 and Tween 80) may improve cyclovirobuxine D accumulation in the brain.

Pashirova et al. developed mixed cationic liposomes based on dihexadecylmethylhydroxyethylammonium bromide to deliver pralidoxime chloride, an acetylcholinesterase reactivator, to treat organophosphorus-induced brain damage [69]. The IN fluorescent-labeled cationic liposomes showed higher rhodamine B absorption in rat brains than the IN and IV solutions and IV liposomes. In rats with paraoxon-induced acetylcholinesterase inhibition, pralidoxime chloride-loaded cationic liposomes reactivated 12% of brain acetylcholinesterase. However, their efficacy was not remarkable. In addition, brain accumulation was not quantitatively determined.

Passoni et al. developed liposomes loaded with cholesterol-D6 to deliver exogenous cholesterol to the brain to treat Huntington’s disease [91]. Following IN-liposome administration, cholesterol-D6 levels in the mice’s striatum, cortex, and cerebellum were approximately 0.4 ng/mg at 24 h and remained stable until 72 h. After 10 IN doses, cholesterol-D6 accumulated similarly (approximately 1.5 ng/mg) in the striatum, cortex, and cerebellum, suggesting the involvement of both olfactory and trigeminal pathways in nose-to-brain delivery. Thus, IN cholesterol-loaded liposome administration could deliver cholesterol to different brain regions for the management of Huntington’s disease.

Butylidenephthalide, a hydrophobic molecule found in *Angelica sinensis*, has anticancer effects. Lin et al. developed liposomes loaded with a butylidenephthalide–(2-hydroxypropyl)-β-cyclodextrin complex [122]. In mice bearing temozolomide-resistant glioblastoma multiforme, IN liposomes showed a longer survival time (60 days) than oral temozolomide (36 days) and oral liposomes (21 days). In addition, drug accumulation in the brain at 30 min following IN liposomes was approximately 10-fold higher than that following oral liposomes.

Baicalin, a compound extracted from *Scutellaria baicalensis*, has a protective effect on some central nervous system diseases, such as Parkinson’s disease, spinal cord injury, and ischemic stroke. Li et al. developed baicalin-loaded liposomes; however, the BBB prevented them from entering the brain effectively [123]. Later, they administered liposomes intranasally for drug delivery to the brain [90]. In rats with middle cerebral artery occlusion, IN liposomes exhibited a 1.5-fold higher in AUC_plasma_ than the IN-free drug. The AUC values of IN liposome-delivered baicalin in the olfactory bulb, hippocampus, striatum, cerebellum, and cortex were 1.6-, 1.1-, 2.1-, 1.1-, and 1.6-fold higher, respectively, than those of the IN-free drug solution. Both IN liposomes and IN-free drug showed high DTE% (1976–3890%) and DTP% (94.9–97.4%), indicating nose-to-brain delivery of baicalin. In pharmacodynamic studies, IN liposomes significantly improved neurological deficits, cerebral infarction, and brain pathological status in rats with middle cerebral artery occlusion.

**Figure 3:**
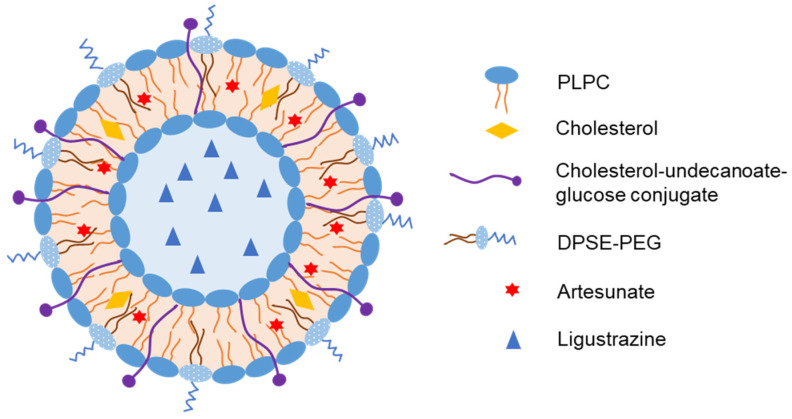
Representative structure of liposomes loaded with artesunate and ligustrazine with cholesterol–undecanoate–glucose conjugate to target glucose transporter 1 at the BBB for treatment of cerebral malaria [37].

Tian et al. prepared liposomes loaded with artesunate and ligustrazine hydrochloride to treat cerebral malaria [37]. They used cholesterol–undecanoate–glucose conjugate to target glucose transporter 1 at the BBB (Figure 3) and found that the brain-targeted liposomes had a longer residence time (up to 48 h) in the mouse brain than the nontargeted liposomes. After IN administration of the liposomes, most of the drug entered the systemic circulation (AUC_plasma_/AUC_brain_ = 442 and 14.9 for artesunate and ligustrazine, respectively); however, AUC_brain/IN_ was still higher than AUC_brain/IV_ for both drugs (4.1- and 1.8-fold, respectively). In addition, a large amount of dihydroartemisinin, an antimalarial metabolite of artesunate, was detected in the brain (AUC_brain,IN/_AUC_brain,IV_ = 4.5). In mice with cerebral malaria, brain-targeted liposomes significantly enhanced the therapeutic effects against cerebral malaria by reducing infection and recurrence rates. Thus, the drugs could enter the brain via both direct and indirect pathways to produce antimalarial effects after IN administration of the liposomes.

Recently, IN-valproic acid-loaded liposomes have been used to effectively prevent epilepsy [124]. The IN liposomes increased the AUC_plasma_ and AUC_brain_ in mice (2.8- and 2.6-fold, respectively) compared to the IN-free drug. Liposomes have also been employed to deliver ferric ammonium citrate to the brain to prevent iron deficiency, which may have neurological consequences [58]. IN administration of liposomes in rats increased iron levels as well as ferritin protein expression in the olfactory bulb, cerebral cortex, and hippocampus. The safety of liposomes was indicated by the absence of apoptosis or abnormal cell morphology in the brain.

Song et al. developed liposomes loaded with indole-3-carbinol, a compound used for lung cancer treatment [125]. Due to its poor oral bioavailability, the efficacy of indole-3-carbinol in clinical trials is limited. Thus, IN liposomes have been developed to enhance the bioavailability of the drug in the lungs as well as its biological effects. The IN liposomes showed 100-fold higher lung exposure of indole-3-carbinol than the oral suspensions and microemulsions in tobacco smoke carcinogen [4-(methylnitro-samino)-1-(3-pyridyl)-1-butanone] (NNK)-treated mice. The IN liposomes also suppressed (by 37%) the formation of O^6^-methylguanine deoxyribonucleic acid (DNA) adduct (a critical DNA adduct in NNK-induced mouse lung tumorigenesis) as well as increased (by 10-fold) the expression of CYP1A1, which is responsible for chemical carcinogen detoxification.

Qiang et al. developed IN liposomes of fexofenadine to prolong drug release and enhance systemic exposure to the drug [44]. Chitosan was coated onto the liposomes to increase mucoadhesion (3-fold) compared to the uncoated liposomes. In rats, the oral free drug exhibited low bioavailability (6.2%). The IN-coated and -uncoated liposomes increased the AUC_plasma_ to 34.7 and 24.5%, respectively. IN chitosan-coated liposomes also exhibited sustained drug exposure in the blood for up to 12 h.

Table 1 summarizes the major features of the above-mentioned small molecule-IN liposomes.

### 3.4. Intranasal Liposomes Containing Therapeutic Peptides and Proteins

Peptides and proteins encapsulated in IN liposomes can be therapeutic peptides and proteins for the treatment of certain diseases or antigen proteins for immunization. In this section, we discuss the former cases.

The H102 peptide (HKQLPFFEED) is a β-sheet breaker peptide that can specifically bind to the β-amyloid monomer, inhibit β-sheet formation, and block the aggregation of the soluble β-amyloid protein. Therefore, it has been used to treat AD. Zheng et al. developed liposomes loaded with this peptide to reduce its degradation and increase its accumulation in the brain following IN administration [40]. In rats, IN liposomes increased the AUC_plasma_ of H102 by 9-fold compared to IN peptide solution. The AUCs of H102 in the olfactory bulb, cerebrum, cerebellum, and hippocampus were 1.7-, 1.7-, 1.6-, and 2.9-fold higher for the IN liposomes than those for the IN-free peptide solution, respectively. In addition, IN liposomes reduced escape latency in rats in the Morris water maze test. The activity of choline acetyltransferase (synthesis enzyme of acetylcholine that ameliorates cholinergic nerve impairment) and insulin-degrading enzyme (a metalloprotease involved in β-amyloid protein degradation) increased in rats treated with IN liposomes, while the levels of acetylcholinesterase decreased.

Glial-derived neurotrophic factor (GDNF) is a protein with potent neurotrophic and regenerative effects on dopaminergic neurons. Migliore et al. have previously developed GDNF-loaded cationic liposomes and evaluated their effects and distribution in the brain following IN administration [92,126]. In both normal and 6-hydroxydopamine-treated (Parkinson’s disease model) rats, the IN liposomes increased dopamine cell number and density of tyrosine hydroxylase staining in the substantia nigra than the IN-protein solution. In addition, IN liposomes significantly increased the GDNF levels in the brain. The autoradiography study revealed that following liposome IN administration, GDNF was distributed along the rat brain’s rostral–caudal axis, confirming the nose-to-brain delivery route.

Luo et al. developed liposomes loaded with nerve growth factor, a protein playing essential roles in the development and maintenance of central sympathetic and sensory neurons [127]. In aging male mice (mice with age-related hypogonadotropic hypogonadism), IN liposomes enhanced sexual hormone secretion, sexual motivation and performance, sperm quality, and fertility.

Ovalbumin is generally used as a model protein to study protein delivery in different systems. Migliore et al. prepared ovalbumin-loaded cationic liposomes for nose-to-brain delivery [84]. Following IN administration of liposomes in rats, Alexa 488–ovalbumin was widely distributed throughout the brain, particularly in the midbrain at 6 h, as shown by fluorescence microscopy. In addition, IN-liposome accumulation increased in the brain and decreased in the stomach and intestine as compared to IN protein accumulation. In another study, ovalbumin was loaded into a cationic liposome-in-thermosensitive gel [18]. The IN-liposome gel showed higher protein accumulation in rat brains at 72 h than the IN-free protein. However, the sample size (*n* = 2) was insufficient to draw any conclusions.

### 3.5. Intranasal Liposomes Containing Nucleic Acids

Liposomes can encapsulate different types of nucleic acids, including DNA, messenger ribonucleic acid (mRNA), and small interfering RNA (siRNA), in their aqueous core. Liposomes and lipid nanoparticles are the most widely used non-viral platforms for gene delivery [128]. Cationic liposomes are generally used to enhance the encapsulation of negatively charged nucleic acids [17].

Zhou et al. developed cationic liposomes loaded with synthetic oligodeoxynucleotides containing CpG motifs (CpG DNA) to prevent pulmonary metastasis [129]. CpG DNA can activate several immune cell types (such as B cells, macrophages, dendritic cells, and natural killer cells) that are critical for antitumor activity. In a mouse pulmonary metastasis model, IN liposomes prevent tumor cell proliferation and prolong survival time. The IN liposomes were mainly distributed in the nose and lungs, whereas the distributions in the liver, kidney, spleen, and omentum were negligible. IN liposomes also enhanced interferon gamma (IFN-γ) production in the lungs. Moreover, the preventive effect of IN liposomes was primarily attributed to the natural killer cell activation [130].

IN liposomes have been developed for delivering nucleic acids to the brain. Dhaliwal et al. prepared cationic liposomes containing GFP mRNA and luciferase mRNA for delivery to the brain via the nose-to-brain route [68]. Successful GFP mRNA delivery to the brain was confirmed by GFP fluorescence. The GFP mRNA expression in the brain following IN liposome was higher than that following IN-free GFP mRNA. Similarly, the IN-liposome luciferase mRNA expressed 12- and 5-fold higher luciferase activity in the cortex and striatum, respectively, than the IN-free form. These results demonstrated that IN administration of cationic liposomes could efficiently deliver mRNA to the brain with minimal systemic exposure.

Vera et al. have recently developed liposomes loaded with two plasmids encoding the CRISPR/Cas9 system and the alpha-L-iduronidase (IDUA) gene targeting the ROSA26 locus for mucopolysaccharidosis type I (MPS I) management [55]. MPS I, or Hurler syndrome, is an autosomal recessive disorder caused by mutations in the IDUA gene that leads to IDUA deficiency. IDUA is responsible for glycosaminoglycan (GAG), dermatan, and heparan sulfate breakdown. Thus, IDUA deficiency results in a high accumulation of GAG dermatan and heparan sulfate in the brain and other organs, which progressively causes multisystemic dysfunction [131]. Plasmids were successfully loaded into liposomes (size: 112 nm; PDI: 0.10), which were desirable for IN administration. A positive charge (+26.4) is crucial to increasing the interaction between the liposomes and the negatively charged mucin sialic groups. Liposomes significantly increased IDUA levels in some brain areas, including the olfactory bulb, frontal cortex, and total cortex, in both short-term (30 days) and long-term (180 days) studies in rats with MPS I. A similar observation was reported in the heart and lungs. In addition, the increased IDUA levels decreased GAG levels in the serum, urine, tissues, and brain cortex, as well as improved behavioral parameters in rats with MPS I [55].

**Figure 4:**
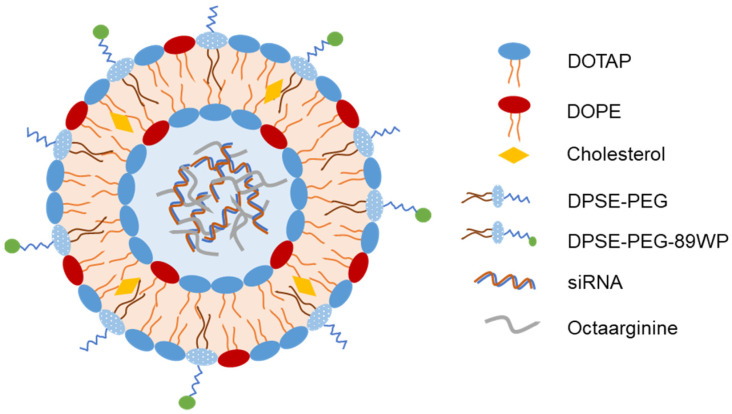
Representative structure of liposomes loaded with c-Myc-targeting siRNA for gene therapy of glioma [41].

Recently, Hu et al. developed liposomes loaded with c-Myc-targeting siRNA for glioma gene therapy via IN administration [41]. The authors employed a cationic peptide octaarginine (R8) to compact siRNA by electrostatic interaction in the liposome core and coated the liposomes with CPP (Q8W, N9W-penetratin (89WP)) to facilitate liposome transport across absorption barriers. DOPE (1, 2-dioleoyl-sn-glycero-3-phosphoethanolamine) played a role as a helper lipid in the siRNA transfection efficiency (Figure 4). The liposomes entered glioma cells by macropinocytosis, which helped prevent their entrapment by lysosomes and subsequently released siRNA into the cytoplasm. In mice, IN 89WP-modified liposomes increased siRNA accumulation in the brain by 4-fold and reduced c-Myc expression level in orthotopic glioma by 2-fold compared to IN 89WP-unmodified liposomes. Finally, IN 89WP-modified liposomes significantly prolonged the survival of glioma-bearing mice. Overall, this liposome system could enhance gene delivery to the brain via IN administration to selectively silence disease-related genes.

Table 2 summarizes the major features of the aforementioned IN liposomes of therapeutic peptides, proteins, and nucleic acids.

## 4. Intranasal Liposomes for Vaccine Delivery

### 4.1. Intranasal Vaccines

The nasal cavity has a large surface area that facilitates antigen absorption. An antigen can be taken up by microfold or dendritic cells and transferred to nasal-associated lymphoid tissue (NALT). Here, the antigen interacts with T and B cells, which can release IgA antibodies or pass through the blood to trigger cellular and IgG-based systemic immunity. Mucosal immunity against respiratory pathogens relies heavily on IgA-mediated protection [132]. FluMist, a live attenuated influenza vaccine, is the only IN vaccine approved by the US FDA (in 2003). In recent years, the IN vaccine has gained significant interest because it is particularly appropriate for various respiratory diseases (such as influenza and COVID-19). IN vaccination is noninvasive, convenient, and applicable to large populations [28]. Different IN vaccines have been developed and evaluated in phase 1 and phase 2 clinical trials [132].

### 4.2. Recent Advances in IN Liposomes for Vaccine Delivery

**Figure 5:**
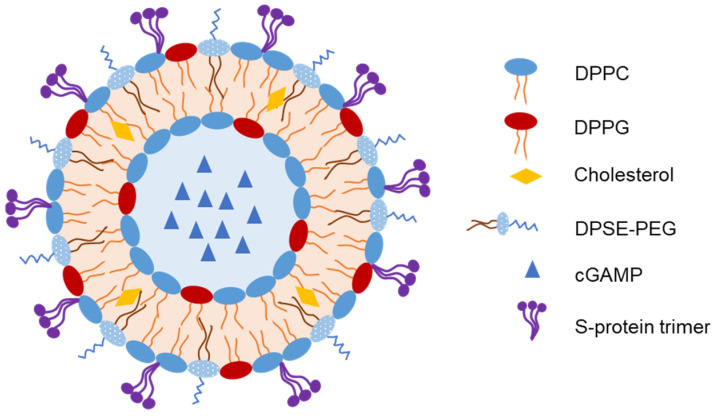
Representative structure of liposomes loaded with spike protein of severe acute respiratory syndrome coronavirus 2 (SARS-CoV-2) as an intranasal (IN) vaccine [73].

Liposomes have been used to encapsulate antigens or vaccine adjuvants to develop IN vaccines. An et al. developed an IN-liposome vaccine to control COVID-19 [73]. They first prepared liposomes containing 2′-3’-cyclic guanosine monophosphate adenosine monophosphate (cGAMP) in the core as adjuvants. The SARS-CoV-2 spike protein (S-protein) trimer was then adsorbed on the liposomes with 61.5% entrapment efficiency (Figure 5). In mice, a single IN-liposome administration elicited serum-neutralizing antibodies comparable to those elicited by other vaccine candidates. The IN-liposome vaccine also increased IgA responses in the nasal compartment and lungs and induced spike-specific T-cell response in the lungs and spleen.

Huang et al. developed liposomes loaded with the SARS-CoV-2 spike glycoprotein receptor-binding domain (RBD) for IN vaccination [133]. Cobalt porphyrin–phospholipids were incorporated into the liposomes for stable RBD binding. They used K18 hACE2 mice expressing the human ACE2 receptor as an animal model. IN liposomes induced RBD-specific IgA production and RBD-specific cellular responses in the lungs. However, IM immunization with the same liposomes further increased RBD-specific IgG antibody levels in the serum. Moreover, both IM- and IN-immunized transgenic mice challenged with SARS-CoV-2 were completely protected against lethal viral infection.

A recent study has reported the development of a liposome vaccine adjuvant, CAF09b, containing the toll-like receptor 3 agonist polyinosinic: polycytidylic acid [134]. IN administration of liposomes to mice upregulates some type I interferon (IFN-I)-related genes. Pretreating mice with IN liposomes prevents death upon lethal challenge with mouse-adapted influenza A H1N1 PR8 virus.

Tada et al. developed cationic liposomes as a mucosal adjuvant for co-administration with pneumococcal surface protein A (PspA) [86]. This IN vaccine induced protective PspA-specific antibodies (nasal, lung, and vaginal IgA; lung and serum IgG; and serum IgG1 and IgG2a) against lethal *S. pneumoniae* inhalation better than IN PspA alone. The survival rate of IN liposome-vaccinated mice after *S. pneumoniae* challenge was 100%, whereas that of the IN antigen-vaccinated mice was <35%. IN liposomes also induced PspA-specific IL-17^+^ T cells (Th17 cells) and increased PspA uptake by nasal dendritic cells. Thus, IN PspA–liposomes may be an efficient vaccine against pneumococcal infection.

Dai et al. developed an IN-liposome vaccine containing the lipopeptide LCP-1 for immunization against Group A *Streptococcus* [75]. Liposomes were coated with polyethylenimine to promote cellular uptake and improve the immune response. In mice, IN liposomes elicited significant systemic and mucosal immune responses. The produced IgA and IgG antibodies effectively opsonized multiple isolates of clinically isolated Group A *Streptococcus*. Yang et al. developed another IN-liposome vaccine against Group A *Streptococcus* by decorating the liposomes with a CPP to enhance their permeability [76]. Among several CPPs evaluated, IN Tat_47–57_ and KALA-coated liposomes induced the highest production of antibodies against Group A *Streptococcus* in mice.

An IN liposome-based vaccine against tuberculosis that contains bacterial T-cell peptides was developed [135]. It induced immune responses and long-lasting central memory responses as well as reduced the bacterial burden and tuberculosis recurrence risk in infected mice. In addition, Bacillus Calmette-Guerin vaccination, followed by IN-liposome immunization, significantly boosted immune responses against tuberculosis via enhanced antigen-specific Th1 and Th17 responses. Another IN-liposome vaccine designed to target CD44 using thioaptamers to boost host immunity against tuberculosis was developed by Singh et al. [74]. In *Mycobacterium tuberculosis*-injected mice with increased lung and spleen loads, IN liposomes preferentially accumulated in the lungs, reduced the bacterial colony-forming units by 10-fold, and increased resident memory T cells. Thus, CD44 thioaptamer-loaded IN liposomes effectively enhanced antitubercular immunity in this mouse model.

Kakhi et al. developed different liposome-based formulations (SUV, MLV, reverse-phase evaporation vesicles, and ultra-flexible liposomes) for use as vaccines with antitumor activity following IN administration [72]. The liposomes were loaded with ErbB2 peptide (a TCD8+ epitope derived from the ErbB2 protein), HA peptide (a TCD4+ epitope derived from influenza hemagglutinin), and lipopeptidic Pam2CAG as an adjuvant. These vaccines were administered intranasally to mice, followed by IV or subcutaneous (SC) implantation of ErbB2-surexpressing cancer cells. Different liposome-based vaccines have shown similar antitumor efficiencies in a lung tumor model. When administered to the SC implantation tumor model, the IN-SUV vaccine resulted in 20% tumor-free mice on day 50, whereas all control mice developed tumors within 8 days after SC implantation of cancer cells; however, the difference was non-significant. Thus, the tumor size and weight should be determined, which may indicate the efficiency of the IN-liposome vaccine.

**Figure 6:**
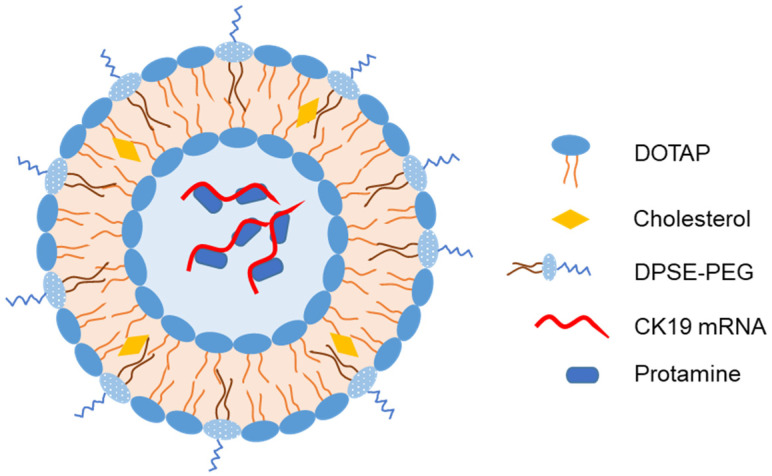
Representative structure of liposomes loaded with mRNA encoding cytokeratin 19 as an intranasal vaccine [71].

Mai et al. developed another IN-liposome vaccine for cancer by loading liposome with mRNA encoding cytokeratin 19, a target antigen for immunotherapy against lung cancer [71]. Protamine was used to compress and completely encapsulate the mRNA in the liposomal core (Figure 6). IN immunization of mice with liposomes elicited a strong cellular immune response via cytokine secretion. More importantly, IN liposomes reduced tumor growth and induced IL-2 and IL-4 secretion in the spleen of Lewis lung cancer model mice.

Abhyankar et al. developed IN liposome adjuvant and combined it with *Entamoeba histolytica* Gal/GalNAc–lectin-derived antigen as a vaccine against diarrheal pathogens [136]. Toll-like receptor agonists were incorporated to enhance the immune response. IN liposomes induced a robust mucosal IgA response as well as systemic and cellular immune responses. In an intestinal amebiasis mouse model, the IN liposomes reduced the infection rate by 55%, and enhanced fecal IgA, serum IgG, systemic IFN-γ, and IL-17A levels, and cleared more than 80% cecal antigen upon challenge [137].

Fan et al. developed hyaluronic acid- and PEG-coated cationic liposomes to deliver a candidate recombinant antigen F1-V for *Yersinia pestis*, a causative agent of pneumonic plague transmitted through pulmonary inhalation, with approximately 100% death rate within a week of infection [78]. The surface decoration of cationic liposomes increased stability, prolonged antigen release (~40% in 3 weeks in vitro), and reduced cytotoxicity in bone marrow-derived dendritic cells by 20-fold. IN liposomes loaded with ovalbumin (a model antigen) and monophosphoryl lipid A (a molecular adjuvant) increased serum IgG and IgG1 levels as well as ovalbumin-specific CD8+ T cells in mice compared with IN antigen plus adjuvant solution. When F1-V and the adjuvant were loaded, the IN liposomes induced 11-, 23-, and 15-fold higher serum F1-V-specific total IgG, IgG1, and IgG2c, respectively, at day 77, compared with the IN antigen plus adjuvant solution. Thus, decorated liposomes are a promising IN vaccine against *Y. pestis* and other infectious pathogens.

Olsen et al. developed a vaccine against *Chlamydia trachomatis* [138]. They first produced recombinant proteins based on the VD4 region from the major outer membrane proteins of *Chlamydia trachomatis*. The antigens were combined with liposome adjuvant CAF01 to induce a strong immune response. In mice, the vaccine reduced the bacterial count in the vagina and prevented pathological changes in the upper genital tract. Mice with simultaneous IM and IN vaccination showed high IgA levels in the vaginal secretions. In the phase 1, first-in-human, double-blind, parallel, randomized, placebo-controlled trial, this vaccine was safe, well-tolerated, and immunogenic; however, the regimen consisted of three IM injections of liposome vaccine followed by two IN administrations of the antigen solution (not containing liposome adjuvant) [139].

Several studies have developed a liposome platform for vaccine delivery and loaded it with a model antigen (such as ovalbumin) to evaluate the immune response. Wang et al. developed galactose-modified liposomes for specific recognition by macrophages and encapsulated ovalbumin for IN immunization [79]. The authors synthesized α- and β-galactosyl lipids and incorporated them into the liposomes. Galactose molecules are recognized by macrophage galactose-type C-type lectins, which increase macrophage uptake and increase tumor necrosis factor-α and interleukin-6 levels. In mice, the IN-modified liposomes induced secretory IgA levels in the nasal and lung wash fluids and a serum IgG antibody response. Moreover, the modified liposomes increased antigen uptake by dendritic cells and induced higher levels of pro-inflammatory cytokines than unmodified liposomes in vitro [140]. Macrophage galactose-type C-type lectins are also expressed on immature dendritic cells in humans and mice, thus mediating modified liposome uptake and initiating the immune response in immature dendritic cells. IN β-galactosylated liposomes resulted in complete protection against EG7 tumor challenge in mice.

Wasan et al. developed cationic liposomes containing ovalbumin as an antigen and incorporated an adjuvant (TriAdj) to boost immune response [141]. IN liposomes induce IgG and IgA production in mice. Tada et al. developed cationic liposomes combined with ovalbumin for IN vaccination [81]. IN liposomes produced IgA in nasal tissues and increased serum IgG1 levels in mice. Cationic liposomes played a role in ovalbumin uptake into dendritic cells in NALT. Then, liposomes were modified with class B oligodeoxynucleotides containing immunostimulatory CpG motifs (CpG ODN), a potent mucosal adjuvant [142]. Loading CpG ODN into cationic liposomes could reduce their dose and cause adverse effects. The IN ovalbumin/CpG ODN/liposomes increased antigen-specific IgA production in mouse nasal mucosa 10-fold compared to the IN non-liposome formulation (only ovalbumin plus CpG ODN). In addition, serum IgG levels in mice vaccinated with IN ovalbumin/CpG ODN/liposomes and IN ovalbumin/liposomes were higher than those in mice vaccinated with IN ovalbumin.

Yusuf et al. developed PEGylated cationic liposomes as carriers for the IN vaccines [77]. These modifications increased the permeability and penetration of liposomes into bovine nasal tissue ex vivo. Serum IgG1, nasal IgA, and vaginal IgA levels in mice vaccinated with IN ovalbumin-loaded liposomes were higher than those in mice vaccinated with IM- and IN-free ovalbumin. Thus, the PEGylated cationic liposome system is a promising IN vaccine delivery system.

Table 3 summarizes the major features of the aforementioned IN-liposome vaccines.

## 5. Author Perspectives and Concluding Remarks

Several issues should be considered when developing IN liposomes for drug, gene, and vaccine delivery. Drug and gene delivery across the BBB for central nervous system diseases and psychiatric disorders remains challenging; however, IN administration has recently emerged as a practical approach for direct nose-to-brain delivery. IN liposomes can effectively improve the drug and gene accumulation in the brain compared to free drugs (oral and IN) or oral formulations. In many cases, the AUC_plasma_ also increased [39,42,83,120,124]. Interestingly, liposomes can cross the BBB and enter the brain after being absorbed into the systemic circulation from the nasal cavity. In some cases, most drugs (>80%) enter the brain via this indirect route [70]. Liposomes can be modified with cholesterol–undecanoate–glucose conjugates to target glucose transporter 1 at the BBB, which allows them to cross the BBB efficiently from the systemic circulation and enter the brain [37]. However, in some cases, most drugs (>90%) enter the brain via direct nose-to-brain transport [90]. Thus, the systemic exposure to IN liposomes and relevant PK parameters for the brain (such as DTE and DTP) should be determined when developing brain-targeted IN liposomes. If the systemic accumulation of drugs is high, the relevant adverse effects should be considered. Inversely, when developing IN liposomes for systemic delivery, encapsulated drugs and genes may accumulate in the brain; thus, relevant central nervous system adverse effects should be assessed [44]. Some studies have demonstrated the efficacy of IN-liposome vaccines in several diseases, such as COVID-19, influenza, tuberculosis, cancer, and diarrhea. The COVID-19 outbreak has facilitated IN vaccine development, and liposomes are a potential formulation approach. Liposomes can encapsulate both antigens and adjuvants to develop IN vaccines. However, IN-liposome vaccines may induce less serum IgG release than IM immunization with the same liposomes [133]. Thus, IN and IM immunizations can be combined to maximize local and systemic immune responses.

The cumulative findings of previous studies have shown that IN liposomes are a promising approach for drug, gene, and vaccine delivery. They can encapsulate many molecules, including small-molecule drugs, peptides, proteins, and nucleic acids, and can bypass first-pass metabolism and degradation by gastrointestinal fluids [108]. In addition, IN administration is needle-free and can be self-administered by patients [89]. Liposomes, particularly those modified with mucoadhesive polymers or loaded in gels, can also protect drugs and genes from enzymatic degradation and mucociliary clearance, increase drug residence time and absorption, and ultimately enhance bioavailability and therapeutic effects [109,115]. In vaccine delivery, IN liposomes induce local and systemic immune responses and appear particularly appropriate for respiratory diseases because they can mimic the natural route of viral infection. In the future, more IN liposomes will be developed to deliver drugs, genes, and vaccines. Mechanistic studies are required to clarify the predominant transport pathways involved in drug and gene delivery, particularly for brain-targeted IN liposomes. Studies should also focus on the adverse effects on untargeted organs. We expect that IN liposomes will soon have clinical applications for systemic and brain delivery. IN-liposome vaccines are also available as a safe and efficient strategy to fight future pandemics. 

## Figures and Tables

**Table 1:** Major features of intranasal liposomes containing small molecules.

Drug	Disease	Composition	Size, PDI, ZP, EE	Primary Outcomes	Year and Ref
Donepezil	Alzheimer’s disease	DSPC, cholesterol, and PEG	102 nm, 0.28, −28 mV, 85%	Higher AUC_plasma_ (2.8- and 1.9-fold) and AUC_brain_ (1.7- and 1.4-fold) vs. oral and IN-free drugs, respectively, in rats.	2016 [39]
Donepezil	Alzheimer’s disease	DPPC, cholesterol, and gel with thiolated chitosan	438 nm, N/A, N/A, 62%	Higher AUC_plasma_ (1.5-fold) and drug content in brain (2.1-fold) vs. oral tablet in rabbits.	2019 [42]
Donepezil	Alzheimer’s disease	HSPC, cholesterol, gel with gellan gum, and xanthan gum	103 nm, 0.108, –30 mV, 93%	Higher permeability than free drug–based gel (6.5-fold); higher AUC_brain_ (2.2-fold), lower AUC_plasma_ (1.4-fold), and liver accumulation (12-fold) than oral solution in rats; reversed the alteration of some biochemical parameters.	2022 [38]
Rivastigmine	Alzheimer’s disease	Egg PC, cholesterol, and DSPE–PEG–CPP	179 nm, 0.333, −8.6 mV, 31%	Increased drug distribution in hippocampus and cortex; reduced acetylcholinesterase and butyrylcholinesterase activities in rats.	2013 [93]
Rivastigmine	Alzheimer’s disease	DSPE–PEG, lecithin, DDAB, and Tween 80	478 nm, 0.56, −8 mV, 48%	Higher AUC_plasma_ (4.2-fold) and AUC_brain_ (4.9-fold) vs. IN-free drug in rabbits.	2017 [43]
Rivastigmine	Alzheimer’s disease	Soya lecithin, and cholesterol	N/A, N/A, N/A, N/A	Rapid onset (Tmax = 5 min), higher AUC_plasma_ (27-fold), reduced clearance rate (2-fold) vs. oral drug solution; rescued the memory deficit in rat behavior studies.	2021 [56]
Galantamine hydrobromide	Alzheimer’s disease	Soya PC, cholesterol, and propylene glycol	112 nm, N/A, −49 mV, 84%	Higher acetylcholinesterase inhibition, C_max,brain_ (3.5-fold) and AUC_brain_ (3.4-fold) vs. oral free drug in rats.	2012 [119]
Celecoxib	Alzheimer’s disease	PC, cholesterol, or erythrocyte membranes	125 nm, 0.122, negative, 98%, 93 nm, 0.104, negative, 90%	Higher brain distribution than IN-free drug suspension improved cognitive decline and reduced β-amyloid deposition in Alzheimer’s disease mice.	2017 [94]
Imatinib mesylate	Alzheimer’s disease	Egg PC, cholesterol, and cardiolipin	102 nm, 0.28, −23 mV, N/A	Higher AUC_brain_ (7-fold) than oral and IN-free drugs in rats.	2021 [57]
Hydroxy-α-sanshool	Alzheimer’s disease	Soybean lecithin (98% PC), and cholesterol	182 nm, 0.207, −54 mV, 73%	Safety; improved learning and memory abilities of mice; protective effects on neuronal cells; higher plasma and brain bioavailability than pure drug (1.7- and 2.1-fold, respectively) in mice.	2022 [83]
Rizatriptan	Migraine	PC and cholesterol glutathione conjugate	194 nm, 0.5, +6 mV, 85%	Higher AUC_plasma_ (1.3- and 2.4-fold) and AUC_brain_ (3.4- and 6.7-fold) vs. IN-non-conjugated liposomes and oral tablet, respectively, in rats.	2020 [120]
Rizatriptan	Migraine	Soya lecithin	149 nm, 0.448, −13 mV, 77%	Higher AUC_plasma_ (4-fold vs. oral solution) in rats.	2022 [121]
Sumatriptan	Migraine	HSPC, cholesterol, and chitosan coating	167 nm, N/A, N/A, 21%	AUC_plasma_: IN chitosan-coated liposomes > IN-uncoated liposomes > IN-free drug solution in rabbits.	2022 [115]
Risperidone	Schizophrenia	Soya PC, cholesterol, and MPEG–DSPE	98 nm, N/A, −28.6 mV, 59%	Higher AUC_brain_ (1.7-, 1.5-, and 1.1-fold for PEGylated, cationic liposomes, and unmodified liposomes, respectively, vs. IV-free drug) in rats; DTE% = 120%, DTP% = 16.7% for PEGylated liposomes.	2016 [70]
Cyclovirobuxine D	Cerebrovascular diseases	Soybean lecithin, cholesterol, DSPE–PEG2000, DSPE–PEG2000–Angiopep-2, and Tween 80	72 nm, 0.301, −15 mV, 92%	Higher in vitro BBB permeation vs. free drug; DTE = 302.22%, DTP = 63.66%, higher AUC_brain_ (2.1- and 1.9-fold vs. IN-free drug and IV liposomes, respectively) in rats	2018 [67]
Pralidoxime chloride	Brain damage by organophosphorus	PC and DHDHAB	80 nm, 0.2, +6 mV, 90%	Higher absorption of rhodamine B in rat brain (vs. IN, IV solution and IV liposomes); reactivated 12% of brain acetylcholinesterase in paraoxon-treated rats.	2018 [69]
Cholesterol	Huntington’s disease	PC and cholesterol-D6	200–300 nm, N/A, N/A, 90%	Cholesterol–D6 evenly distributes and accumulates in mouse brain.	2020 [91]
Butylidenephthalide	Glioblastoma multiforme	DMPC, cholesterol and drug-cyclodextrin complex	360 nm, 0.53, N/A, 98%	Longer median survival time of mice bearing drug-resistant glioblastoma multiforme (60 days vs. 21 days of oral liposomes); higher drug level in brain after 30 min (10-fold vs. oral liposomes).	2020 [122]
Baicalin	Cerebral ischemia stroke	Soybean lecithin and cholesterol	160–190 nm, N/A, −5.7 mV, 42%	In rats with middle cerebral artery occlusion, the IN liposomes exhibited. Higher AUC_plasma_ (1.5-fold), AUC_olfactory bulb_ (1.6-fold), AUC_hippocamus_ (1.1-fold), AUC_striatum_ (2.1-fold), AUC_cerebellum_ (1.1-fold), AUC_cortex_ (1.6-fold) vs. IN-free drug; high DTE% (1976–3890%) and DTP% (94.9–97.4%); improved neurological deficits, cerebral infarction, and brain pathological status in rats with middle cerebral artery occlusion.	2020 [90]
Artesunate and ligustrazine	Cerebral malaria	PLPC, cholesterol–undecanoate–glucose conjugate and DSPE–PEG	86 nm, N/A, N/A, 90% and 30%	Longer drug residence in brain; higher AUC_brain_ than IV-free drug; reduced infection rate and recurrence rate in mice with cerebral malaria.	2022 [37]
Valproic acid	Epilepsy	PC and cholesterol	92 nm, 0.21, −43 mV, 85%	Higher AUC_plasma_ (2.8-fold) and AUC_brain_ (2.6-fold) in mice vs. IN-free drug.	2022 [124]
Ferric ammonium citrate	Iron deficiency	Lecithin and cholesterol	40 nm, N/A, −48 mV, 97%	Increased brain iron and ferritin expression compared with IN-free drug; no apoptosis or changes of cell morphology in rat brain.	2017 [58]
Indole-3-carbinol	Lung cancer	DPPC and cholesterol	664 nm, 0.277, −6.7 mV, 7.9%	Higher lung exposure (100-fold) vs. oral suspensions/microemulsions; reduced O^6^-methylguanine DNA adduct; increased (10-fold) CYP1A1 expression in mice with lung cancer.	2014 [125]
Fexofenadine	Allergic rhinitis	DPPC, DPPG, cholesterol, and chitosan coating	716 nm, 0.1, +11.8 mV, 66%	Higher mucoadhesion (3-fold) vs. uncoated liposomes; higher AUC_plasma_ (5-fold) vs. oral free drug; sustained drug exposure up to 12 h.	2012 [44]

AUC, area under the concentration–time curve; BBB, blood–brain barrier; C_max_, maximum drug concentration; CPP, cell-penetrating peptide; DDAB, didecyldimethyl ammonium bromide; DHDHAB, dihexadecylmethylhydroxyethylammonium bromide; DMPC, 1,2-dimyristoyl-sn-glycero-3-phosphocholine; DPPC, 1,2-dipalmitoyl-sn-glycero-3-phosphocholine; DPPG, 1,2-dipalmitoyl-sn-glycero-3-phospho-(1′-rac-glycerol); DSPC, 1,2-distearyl-sn-glycero-3-phosphocholine; DSPE–PEG, 1,2-distearoyl-sn-glycero-3-phosphoethanolamine-N-[amino (polyethylene glycol)]; HSPC, hydrogenated soy phosphatidylcholine; IN, intranasal; IV, intravenous; MPEG–DSPE, methoxy-PEG–DSPE; N/A, not available; PLPC, 1-palmitoyl-2-lauroyl- sn-glycero-3-phosphocholine; and PC, phosphatidylcholine.

**Table 2:** Major features of intranasal liposomes of therapeutic peptides, proteins, and nucleic acids.

Peptide/Protein/Nucleic Acid	Disease	Composition	Size, PDI, ZP, EE	Primary Outcomes	Year and Ref
H102 (β-sheet breaker peptide)	Alzheimer’s disease	Egg PC, DSPE–PEG, and cholesterol	112 nm, 0.185, −3 mV, 71%	Higher AUC_olfactory bulb_ (1.7-fold), AUC_cerebrum_ (1.7-fold), AUC_cerebellum_ (1.6-fold), AUC_hippocamus_ (2.9-fold), ameliorated spatial memory impairment, decreased acetylcholinesterase activity, increased choline acetyltransferase and insulin-degrading enzyme activity in rats (vs. IN-free peptide solution).	2015 [40]
GDNF	Parkinson’s disease	DOPC, cholesterol, and stearylamine	149 nm, N/A, +30 mV, 95%	Increased dopamine cell number and density of tyrosine hydroxylase staining in substantia nigra; increased GDNF level in rat brain.	2014-2015 [92,126]
Nerve growth factor	Hypogonadotropic hypogonadism	Soybean lecithin and cholesterol	N/A, N/A, N/A, N/A	Enhanced sexual function, sperm quality, and fertility in aging male mice.	2018 [127]
Ovalbumin (model protein)	None	DOPC, cholesterol, stearylamine	299 nm, N/A, +19 mV, 94%	Higher protein brain accumulation and lower stomach and intestines accumulation vs. IN protein solution.	2010 [84]
Ovalbumin (model protein)	None	DPPC, DOTAP, and cholesterol,	221 nm, 0.2, +22.7 mV, 85%	Higher protein accumulation in rat brain.	2018 [18]
CpG DNA	Pulmonary metastasis	DOTMA, DOTAP, and cholesterol	N/A, N/A, N/A, N/A	In a mouse pulmonary metastasis model: reduced proliferation of tumor cells; prolonged survival time; mainly distributed in nose and lung; higher interferon gamma production in lungs.	2010 [129]
Luciferase mRNA	Neurodegenerative diseases	DPPC, DOTAP, and cholesterol	195 nm, 0.19, +36 mV, 80%	Higher luciferase activity (12- and 5-fold in the cortex and striatum, respectively) vs. IN-free luciferase mRNA.	2020 [68]
Plasmid for the IDUA gene targeting the ROSA26 locus	Mucopolysaccharidosis type I	DOPE, DOTAP, and DSPE–PEG	112 nm, 0.10, +26 mV, N/A	Increased IDUA levels in brain and organs; reduced GAG levels in serum, urine, tissues, and brain cortex; improved behavioral parameters.	2022 [131]
c-Myc-targeting siRNA	Glioblastoma	DOTAP, DOPE, cholesterol, and 89WP-PEG2000-DSPE	<130 nm, N/A, N/A, N/A	Increased siRNA accumulation in the brain (4-fold) and reduced c-Myc expression (2-fold) vs. unmodified liposomes; prolonged the survival time of glioma-bearing mice.	2022 [41]

AUC, area under the concentration–time curve; CpG DNA, synthetic oligodeoxynucleotides containing CpG motifs; DOTMA, N-[1-(2,3-dioleyloxy)propyl]-N,N,N-trimethylammonium chloride; DOTAP, 1,2-dioleoyl-sn-glycero-3-trimethylammonium propane; DOPE, 1, 2-dioleoyl-sn-glycero-3-phosphoethanolamine; DOPC, dioleoylphosphatidylcholine; DPPC, 1,2-dipalmitoyl-sn-glycero-3-phosphocholine; DSPE–PEG, 1,2-distearoyl-sn-glycero-3-phosphoethanolamine-N-[amino (polyethylene glycol)]; GAG, glycosaminoglycan; GDNF, glial-derived neurotrophic factor; IDUA, alpha-L-iduronidase; IN, intranasal; N/A, not available; and PC, phosphatidylcholine.

**Table 3:** Major features of intranasal liposomes for vaccine delivery.

Loading Agent	Disease/Pathogen	Composition	Size, PDI, ZP	Primary Outcomes	Year and Ref
SARS-CoV-2 S-protein trimer	COVID-19	DPPC, DPPG, cholesterol, DPPE–PEG, and cGAMP	105 nm, 0.24, −30 mV	Systematic and mucosal immune responses in mice; spike-specific IgA responses in nasal compartment and lungs.	2021 [73]
SARS-CoV-2 RBD spike glycoprotein	COVID-19	DOPC, cholesterol, CoPoPs, and 3D6A–PHAD	N/A, N/A, N/A	Induced RBD-specific IgA production and RBD-specific cellular responses in the lungs; full protection against lethal virus in K18 hACE2 transgenic mice.	2022 [133]
None	Influenza virus disease	DDA, MMG, and polyinosinic: polycytidylic acid	150–200 nm, N/A, +40 mV	Upregulation of some IFN-I-related genes; reduced mice death.	2022 [134]
Pneumococcal surface protein A (PspA)	*Streptococcus pneumoniae*	DOTAP, and DC-chol	138 nm, N/A, +4 mV	Higher antibodies in nasal, lung, and vagina vs. IN protein alone; survival rate = 100% in infected mice; induced PspA-specific IL-17^+^ T cells (Th17 cells); increased uptake of PspA by nasal dendritic cells.	2018 [86]
Lipopeptide LCP-1	Group A Streptococcus	DPPC, cholesterol, and polyethylenimine	138 nm, 0.09, +39 mV	Elicited production of IgA and IgG antibodies.	2020 [75]
Lipopeptide LCP-1	Group A Streptococcus	DPPC, cholesterol, and CPP	112 nm, 0.08, +38 mV	IN Tat_47–57_ and KALA-coated liposomes resulted in the highest production of antibodies against Group A *Streptococcus* in mice.	2021 [76]
T-cell peptides	Tuberculosis	N/A	N/A, N/A, N/A	Induced immune responses and long-lasting central memory responses; reduced bacterial burden and risk of recurrence in infected mice	2021 [135]
CD44 thioaptamer	Tuberculosis	Soybean PC, cholesterol, and DSPE–PEG	205 nm, N/A, −23 mV	Accumulated in lungs; reduced bacterial colony-forming units; increased resident memory T cells in *Mycobacterium tuberculosis*-injected mice.	2022 [74]
ErbB2 peptide and HA peptide	Cancer	Egg PC, L-α-phosphatidylglycerol, and cholesterol	68 nm, 0.2, −82 mV	Efficient antitumor efficiency in lung tumor model for all different liposome-based vaccines.	2016 [72]
mRNA-encoded CK19	Lung cancer	DOTAP, cholesterol, DSPE-–PEG, and protamine	170 nm, 0.18, +10 mV	Elicited cytokine secretion; reduced tumor growth in tumor-bearing mice.	2020 [71]
LecA protein	Diarrhea	DPPC, DPPE–PEG, and cholesterol	65–130 nm, N/A, N/A	Induced mucosal, systemic, and cellular immune response; reduced infection rate by 55%; enhanced fecal IgA, serum IgG, systemic IFN-γ, and IL-17A; cleared > 80% cecal antigen upon challenge.	2017–2018 [136,137]
Recombinant antigen F1-V	*Yersinia pestis*	DOTAP, DOPE, hyaluronic acid, and PEG	N/A, N/A, N/A	Prolonged release of antigen; increased stability; higher serum F1-V-specific IgG, IgG1, and IgG2c at day 77 (11-, 23-, and 15-fold vs. IN solution, respectively).	2015 [78]
VD4-based recombinant proteins	*Chlamydia trachomatis*	DDA and TDB	N/A, N/A, N/A	Reduced bacterial numbers in vagina; prevented pathological changes in upper genital tract; higher IgA levels in vaginal secretions in mice with simultaneous IM and IN vaccination.	2015 [138]
Ovalbumin	None	PC, cholesterol, and β-galactosyl DLPE	~1000 nm, 0.221, −14 mV	Higher IgA (nasal and lung) and IgG (serum); complete protection against EG7 tumor challenge in mice.	2013–2015 [79,140]
Ovalbumin	None	Egg PC, DDAB, and DOPE	<200 nm, 0.36, +50 mV	IgG and IgA production.	2019 [141]
Ovalbumin	None	DOTAP and DC-chol	57 nm, N/A, +9 mV	The IN liposomes produced IgA in nasal tissues and increased serum IgG1 levels in mice.	2015 [81]
Ovalbumin	None	DOTAP, DC-chol, and class B CpG ODN	138 nm, N/A, +4 mV	Higher IgA in mouse nasal mucosa (10-fold) vs. IN nonliposome formulation; higher serum IgG vs. IN ovalbumin.	2017 [142]
Ovalbumin	None	Soybean PC, and DDA, TPGS	109 nm, N/A, +50 mV	Higher serum IgG1, nasal IgA, and vaginal IgA levels in mice (vs. IM- and IN-free ovalbumin).	2017 [77]

3D6A–PHAD, monophosphoryl hexa-acyl lipid A and 3-deacyl; cGAMP, 2′-3″cyclic guanosine monophosphate adenosine monophosphate; CoPoPs, cobalt porphyrin–phospholipids; COVID-19, coronavirus disease 2019; CPP, cell-penetrating peptide; CpG ODN, oligodeoxynucleotide containing immunostimulatory CpG motifs; DC-chol, cholesteryl 3β-N-(dimethylaminoethyl) carbamate; DDA, dimethyldioctadecylammonium bromide; DDAB, didecyldimethyl ammonium bromide; DLPE, 1,2-didodecanoyl-sn-glycero-3-phosphoethanolamine; DOPE, 1,2-dioleoyl-sn-glycero-3-phosphoethanolamine; DOTAP, 1,2-dioleoyl-3-trimethylammoniopropane; DPPC, 1,2-dipalmitoyl-sn-glycero-3-phosphocholine; DPPG, 1,2-dipalmitoyl-sn-glycero-3-phospho-(1′-rac-glycerol); DPPE–PEG, dipalmitoyl-sn-glycero-3-phosphoethanolamine-N-[methoxy(polyethylene glycol)]; DSPE–PEG, 1,2-distearoyl-sn-glycero-3-phosphoethanolamine-N-[amino (polyethylene glycol)]; HA peptide, influenza virus hemagglutinin-derived peptide; IFN, interferon gamma; Ig, immunoglobulin; IL, interleukin; IM, intramuscular; IN, intranasal; MMG, monomycoloyl glycerol; N/A, not available; PC, phosphatidylcholine; RBD, receptor-binding domain; SARS-CoV-2, severe acute respiratory syndrome coronavirus 2; TDB, trehalose 6,6-dibehenate; and TPGS, d-alpha-tocopherol polyethylene glycol 1000 succinate.

## Data Availability

Not applicable.
